# Supplementary material for: Mapping the evolution of fertility support policies in China: A content and instrumental analysis
Source: PLoS One. 2025 Oct 9;20(10):e0332137. doi: 10.1371/journal.pone.0332137 (PMC12510515; doi:10.1371/journal.pone.0332137)
Supplement: S1 Appendix — (ZIP) [file pone.0332137.s001.zip › S1 Appendix. 226 original policy documents/121-商务部关于印发《居民生活服务业发展“十三五”规划》的通知(FBM-CLI-4-288097).doc]

商务部关于印发《居民生活服务业发展“十三五”规划》的通知

**发布部门：** [商务部](https://www.pkulaw.com/law/chl?Aggs.IssueDepartment=60220) [机构沿革](javascript:void(0))

**发文字号：**商服贸发〔2016〕488号

**发布日期：**2016.12.27

**实施日期：**2016.12.27

**时效性：** [现行有效](https://www.pkulaw.com/law/chl?Aggs.TimelinessDic=01)

**效力级别：** [部门工作文件](https://www.pkulaw.com/law/chl?Aggs.EffectivenessDic=XE0303)

**法规类别：** [商贸物资综合规定](https://www.pkulaw.com/law/chl?Aggs.Category=07501)

商务部关于印发《居民生活服务业发展“十三五”规划》 的通知
（商服贸发〔2016〕488号）

各省、自治区、直辖市、计划单列市及新疆生产建设兵团商务主管部门，部机关各单位，各特派员办事处，各直属事业单位，各商会、协会、学会：
　　为促进居民生活服务业在“十三五”期间健康快速发展，充分发挥居民生活服务业在稳增长、调结构、惠民生、促就业等方面的重要作用，根据《[中华人民共和国国民经济和社会发展第十三个五年规划纲要](https://www.pkulaw.com/chl/3fd7b48d6cb8d951bdfb.html)》、《商务发展第十三个五年规划纲要》和《国内贸易流通“十三五”发展规划》，商务部制定了《居民生活服务业发展“十三五”规划》，现印发给你们，请结合工作实际认真贯彻执行。

商务部
2016年12月27日

　　居民生活服务业发展“十三五”规划

　　餐饮、住宿、家政、洗染、沐浴、美容美发、家电维修、人像摄影等居民生活服务业是保障和改善民生的重要行业，对稳增长、调结构、促就业等具有重要意义。为促进居民生活服务业持续健康发展，更好的满足城乡居民服务消费需求，根据党中央、国务院的总体部署和“十三五”时期我国居民生活服务业发展面临的新形势，制定本规划。
　　**一、**发展基础
　　“十二五”期间，各地积极采取措施促进居民生活服务业较快发展，有效发挥了居民生活服务业对经济稳定增长和转型升级的促进作用，为实现经济社会发展预期目标作出了重要贡献。
　　（一）服务消费快速增长。
　　据商务部商贸服务典型企业统计数据测算，2015年，我国居民生活服务业营业收入为5.2万亿元，比上年增长12.6%，高于国内生产总值增速。大众化需求占主导地位，餐饮、住宿、家政等服务逐渐成为百姓的习惯性消费，如大众化餐饮占餐饮市场的80%，住宿消费中经济型酒店发展迅速，近40%的城镇家庭需要家政服务。个性化需求爆发式增长，随着互联网技术的广泛应用，以“80、90后”为代表的消费群体追求时尚，个性化需求层出不穷。专业化需求增长迅速，随着老龄化进程的加快，以突出技能为特征的养老、健康等服务需求呈现旺盛态势。体验式、特色化服务需求旺盛，养生保健、美体健身、休闲娱乐等体验式服务已成为居民生活服务消费的常态，海鲜餐厅配以海洋、沙滩为主题的用餐环境受到消费者欢迎。
　　（二）服务供给日益丰富。
　　信息技术全面融入生活服务，团购型、体验型、共享型、上门服务型等O2O模式在餐饮、家政、美容美发等生活服务领域得到广泛应用，方便、快捷、安全、舒适的服务模式不断推陈出新。餐饮业出现厨师上门等服务，住宿业中主题酒店、民宿短租发展迅猛，家政服务业在传统小时工、病患陪护基础上拓展了上门洗车、生活用品配送等服务，美容美发业出现头皮护理、接发、美甲、美睫等服务。沐浴业在传统洗浴、足浴保健、温泉水疗等基础上推出医疗养生等服务。
　　（三）服务方式不断创新。
　　智慧服务、融合服务、聚集服务、品质服务、精准服务、安全服务已经成为居民生活服务业发展的大趋势。信息技术和智能设备的运用更加广泛，智慧服务的范围不断扩大、水平不断提高。线上交易与线下服务的融合更加紧密，不同业态、不同行业之间的融合进一步深化，融合服务更加普遍。社区生活综合服务中心、购物中心和乡镇服务综合体将集中向居民提供各类生活服务，集聚服务更加广泛。顺应消费者需求方式的变化，一站式、精细化的品质服务越来越普及。顺应消费者注重体验、崇尚品位的个性化需求，精准服务将成为普遍追求。老百姓对人身、健康、财产等方面安全的要求越来越高，安全服务将成为共识。
　　（四）服务质量稳步提升。
　　居民更加注重生活服务的内在体验、便捷程度和服务水平。质优价廉的服务成为主流，从业人员素质不断提升，行业法规不断健全，服务流程不断规范，信用评价体系逐步建立，消费者保护机制日益完善，优质服务供给的基础逐步夯实。舒适便利的服务成为普遍要求，居民生活服务业基础设施投入不断加大，信息技术的运用水平、消费便利化和舒适化程度不断提高。
　　同时，居民生活服务业也存在以下问题，一是总量不足，大众化早餐等便民服务网点缺失，家政服务供给严重不足，难以满足市场需求；二是结构不优，居民生活服务企业组织化程度低，连锁化水平不高，低端生活服务供给较多，除餐饮、住宿外，高档、高端、有品质的生活服务供给不足；三是质量不高，服务标准不健全，诚信水平不高，从业人员大多缺乏专业培训，服务不规范、不安全；四是负担过重，房租、人员成本不断上升，税费负担较重，企业经营压力较大。

　　**二、**总体要求
　　（一）指导思想。
　　全面贯彻党的十八大和十八届三中、四中、五中、六中全会精神，以马克思列宁主义、毛泽东思想、邓小平理论、“三个代表”重要思想、科学发展观为指导，深入贯彻习近平总书记系列重要讲话精神，紧紧围绕统筹推进“五位一体”总体布局和协调推进“四个全面”战略布局，牢固树立和贯彻落实新发展理念，以满足人民群众日益增长的生活服务需求为出发点，着力推进供给侧结构性改革，提高供给能力、优化供给结构、提升供给质量、改善供给环境、规范供给秩序，坚持创新驱动，促进居民生活服务业信息化、标准化、集约化发展，加快构建多层次、全方位的居民生活服务体系，扩大服务消费，促进消费结构升级。
　　（二）基本原则。
　　坚持市场主导和政府引导相结合。充分发挥市场在资源配置中的决定性作用，促进要素自由流动，调动各类市场主体参与的积极性。更好地发挥政府作用，完善政策促进体系，营造良好的发展环境，引导居民生活服务企业加快转变发展方式，创新发展模式，提升发展能力。
　　坚持重点突破和统筹协调相结合。针对居民生活服务业发展的主要矛盾和薄弱环节，创新支持方式，探索新的发展途径，着力解决制约居民生活服务业发展的突出问题。因地制宜，分类指导，统筹推进居民生活服务业区域、城乡、行业和业态间协调发展。
　　坚持保障基本和多元发展相结合。以保障居民基本生活服务供给为重点，鼓励各类市场主体提供安全、便捷、实惠、绿色的大众化服务，支持居民生活服务企业集聚式发展，加快线上线下融合发展，培育新型、特色服务模式，健全居民生活服务体系。
　　（三）发展目标。
　　以需求为导向，增加有效供给，到2020年，初步形成优质安全、便利实惠、城乡协调、绿色环保的城乡居民生活服务体系，更好地适应人民群众大众化、多元化、优质化的消费需求。
　　服务能力明显增强。城市社区便民服务设施基本全覆盖，农村地区便民服务体系初步形成，家庭生活服务更为便利，大众化生活服务更为普及，各种服务业态全面发展。
　　市场主体持续壮大。居民生活服务市场主体全面发展，信息化、标准化、集约化水平显著提升，形成一批有较强影响力的品牌企业和众多富有创新活力的中小企业，居民生活服务从业人数占全部就业人数的比重有所提高。
　　发展环境不断优化。法规标准体系逐步健全，监管机制逐步完善，政策促进体系逐步建立，规划布局更加合理，质量体系更加规范，诚信体系建设得到加强，形成社会各方积极参与的良好氛围。

　　**三、**主要任务
　　（一）以供给侧结构性改革为主线全面提升供给能力。
　　1. 优化供给结构。
　　做优基本服务。以满足家庭需求为着力点，鼓励各类市场主体向居民家庭提供日常生活用品和餐饮、家政等服务。着力保障家政服务需求，充分发挥已建家政服务网络中心的作用，鼓励家政服务企业和妇女组织、扶贫办等机构共同搭建家政服务员输出输入对接平台，引导农村富余劳动力从事家政服务，保障养老服务、护幼服务需求。通过制度化管理、标准化服务、连锁化发展、规范化经营，推动企业专业化、规模化、品牌化发展，探索发展员工制家政服务企业的新途径；积极探索以市场化方式推动养老服务产业发展，推动卫生服务与家政护理协同发展，推动构建居家养老、社区养老等大众化养老服务体系。
　　做强大众化服务。以方便快捷、经济实惠为目标，鼓励生活服务企业提供面向大众的服务，创新服务形式，丰富服务内容。着力发展大众化餐饮，引导餐饮企业建立集中采购、统一配送、规范化生产、连锁化经营的生产模式，鼓励高端餐饮企业发展大众化餐饮网点和品牌，优先提供平价特色产品；支持餐饮企业在社区、学校、医院、办公集聚区、交通枢纽等地设立经营网点，着力发展营养、卫生、美味、经济的快餐和风味小吃，大力发展早餐、快餐、团餐、特色小吃、食街排挡等民生服务业态。
　　做好农村生活服务。加快发展农村生活服务业，健全农村生活服务网络，扩大农村服务消费。针对小城镇、城郊结合部、旅游景区、偏远农村等地区，结合本地实际情况引导各类市场主体参与农村居民生活服务体系建设。顺应新型城镇化趋势，结合地区特点和特色乡镇优势，积极发展农家乐、客栈民宿、温泉养生等多样化、特色化服务，完善生活服务配套功能。发挥中心城市的引领作用，鼓励各类市场主体进一步向乡镇、农村延伸服务网络，建立完善适合农村地区特点的购物、餐饮、理发、废旧物品回收等服务体系。
　　做精专业化服务。顺应消费升级趋势，以满足专业化需求为导向，通过强化技能培训、引进国外高素质劳动力等方式，促进行业向更高形态、更宽领域发展，满足高品质服务需求。完善休闲娱乐设施，提供面向年轻消费群体追求时尚、注重体验、崇尚品位的服务，鼓励开发面向老人、中小学生、病人等特定消费群体的产品，着力发展地方特色餐饮、休闲养生等品质化服务。

专栏1 提升供给能力

实施家政扶贫行动。在贫困地区建设家政服务人员供应基地，开展“公司＋基层组织”和“基层组织＋家庭”的定向对接，加大对贫困地区妇女入职培训的扶持力度，依托龙头企业、培训机构、行业协会及社会团体开展职业道德、实际操作能力等方面的教育。

实施农村服务消费促进工程。制定出台财政、金融等支持措施，培育一批集零售、文化、生活等多种功能于一体的乡镇商贸中心，鼓励农资超市提供农机租赁、维修、废旧物资回收等服务，支持有条件的乡镇建设综合生活服务中心，集中提供理发、维修、衣物织补、照看幼儿、帮工、信息中介、废旧物资回收等服务。

　　2.扩大供给能力。
　　完善服务设施，合理配置社区餐饮网点、社区菜店、再生资源回收点等大众化社区服务设施，在城郊结合部、乡镇、农村建设符合当地民俗习惯的生活服务站，大力发展微利性居民生活服务。健全服务网络，以普通大众为主要服务对象，加快构建以家庭为基础、社区为依托、企业为主体的居民生活服务体系。丰富服务内容，鼓励各类市场主体在提供零售、餐饮、快递、维修、家政、养老、健康、婴幼儿看护等基本生活服务的基础上，以满足个性化、专业化需求为导向，提供不同层次的多样化服务。
　　3.推动转型发展。
　　加快法规标准建设，健全体制机制，倡导节约消费，鼓励社会资本积极参与大众化餐饮服务体系建设；以创建绿色饭店为抓手促进绿色发展，引导支持住宿新业态健康发展；在保障基本服务需求的同时，积极开发高端家政服务；推进人像摄影与相关产业融合发展，大力推广沐浴养生的健康理念，完善家电维修、美容美发、洗染等服务质量管理体系，增强企业发展能力和服务供给能力。

专栏2 健全生活服务市场体系

实施居民生活服务业转型发展行动计划。推动居民生活服务企业适应经济新常态和居民消费需求变化，推进便利化发展，最大限度地保障市场供应；加快线上线下融合创新发展，推动行业转变发展方式；推动体制机制创新，加强法规、标准等制度建设，健全信用体系，着力解决传统居民生活服务企业管理方式粗放、创新能力不足、服务质量不高等问题。

　　（二）以集约化为导向提升组织化程度。
　　1.提高经营效率。
　　提升连锁化水平，以规模化经营、规范化管理和专业化服务为核心，加快发展直营连锁，规范发展特许连锁，引导发展自愿连锁。提升品牌影响力，鼓励居民生活服务企业加大品牌建设投入，增强自主创新能力，开发自主品牌，扩大品牌影响力，重点打造一批品牌企业。提升规模化水平，引导优势企业通过参股、联合、合作等方式，跨地区整合资源，创新完善物流配送体系，实现集中配送，标准化管理，培育运作规范、竞争力强的大型企业（集团）。提升产业链效益，向产业链的上下游延伸，建设网络化、智能化产业链，健全居民生活服务供需对接、信息咨询等功能，系统性降低产业链成本。
　　2.降低经营成本。
　　加快推动居民生活服务业集聚式发展。鼓励有实力的企业和社会组织参与社区服务体系建设，整合社区便民服务资源，向社区居民提供全方位的便民服务，逐步形成快捷、放心、满意的社区居民生活服务体系。广泛吸纳社区服务企业和社会组织信息资源，加强电子商务企业与社区商业网点融合互动，拓展社区公共服务综合信息平台的服务功能，完善社区生活服务业态配置，增强社区服务企业在幼儿日间照料和居家养老等方面的服务功能，推动社区生活服务专业化、便利化发展。结合各地区产业特色和历史人文特点，通过科学规划、市场化运作，培育一批特色鲜明的生活服务街区。

专栏3 完善社区服务体系

建设社区生活综合服务中心。集中提供零售、餐饮、家政、养老、洗染、美容美发、维修、寄存、快递收取、金融、文化、休闲、再生资源回收等服务，完善“一站式”服务功能，增强社区生活综合服务能力。

　　3.推动绿色发展。
　　引导支持居民生活服务企业加快设施设备的节能环保改造，加强废旧物品综合利用，将节能降耗落实到生产经营的各个环节，培育绿色服务企业。完善法规标准，支持住宿企业减少使用一次性用品，推进绿色饭店创建工作。鼓励餐饮企业分类回收生活垃圾，加强餐厨废弃物回收利用。积极向沐浴、洗染、理发等用水量较大的行业推广普及节水、节能技术和设备。建立节俭消费提醒提示制度和激励机制，加强宣传引导，倡导消费者使用节能节水和环境友好产品，鼓励绿色、低碳、健康消费。

专栏4 绿色促进

创建绿色饭店。加大《绿色饭店》国家标准的宣贯工作力度，完善绿色饭店评价办法，指导行业中介组织加强绿色饭店评审员队伍建设，提升绿色饭店创建工作质量，鼓励饭店企业对照明、空调、锅炉系统进行节能改造，节约用电、用水、用气，减少各类用品过度豪华包装，为顾客提供舒适、安全、利于人体健康的绿色客房和绿色餐饮。

　　（三）以信息化为手段提升创新能力。
　　1.运用信息技术。
　　建设信息管理系统，鼓励居民生活服务企业运用移动互联网、物联网等现代信息技术，加强对服务人员、服务流程、服务标准等关键环节和要素的管理，挖掘内部潜力，提高经营效益。实现服务模式再造，支持大数据技术在居民生活服务领域的应用，引导企业完善线上支付、信息交互等功能，提升线下体验、配送和售后等服务水平。促进共享发展，引导居民生活服务企业和第三方机构建立信息服务平台，建立政府与社会紧密互动的数据采集机制，对接供求信息、跟踪服务质量、处理服务纠纷，加快构建大数据产业链，推动家政、餐饮等生活服务业与健康服务协同发展。
　　2.促进融合发展。
　　着力推动“互联网＋生活服务”的全面融合发展，改造提升客户关系管理、在线交易等功能，实质性地创新服务消费方式。鼓励餐饮企业采用半成品配送、厨师上门等方式实现创新发展。支持住宿企业利用互联网建立直接销售体系，充分利用第三方在线预订平台拓展营销渠道，加快发展客栈民宿、租赁式公寓等新型业态。鼓励发展集聚家政服务企业的信息平台，引导各类家政服务信息平台提供面向老人的在线咨询、法律援助、健康保险、医疗护理等服务。支持洗染、维修、美容美发等行业依托已有电商平台或自有服务平台，开展上门取送、到家服务等业务。

专栏5 服务创新

实施“互联网＋生活服务”行动。鼓励有条件的地区设立促进居民生活服务业创新发展基金，通过互联网股权众筹等方式，开展居民生活服务业线上线下融合创新发展试点和示范工作，推动居民生活服务企业创新发展。

发展租赁式公寓。推动有关部门制修定消防、治安管理等法律法规，完善环境卫生、服务流程、商业信用等规范，在有效管理的同时促进行业有序发展。鼓励互联网平台完善点评机制和惩戒机制，引导行业中介组织完善标准，加强行业自律，提升服务水平。

　　（四）以标准化为目标提升服务质量。
　　1.加强标准建设。
　　健全标准体系，重点围绕诚信建设、服务质量、业态创新等方面制修订标准，对企业行为、商业信用、服务流程等方面提出要求，加快推动企业标准向国家标准、行业标准转化，构建国家标准、行业标准、地方标准与企业标准相互配套、相互补充的标准体系，增强标准适用性，提高标准质量。强化标准实施，建立政府引导、社会中介组织推动、骨干企业示范应用的标准实施应用机制，加强对现有标准实施情况的监督检查和跟踪评价。完善标准管理，优化标准制修订程序，提高标准立项和发布效率，推动标准查询、立项、制订、修订、审批、发布等在线化管理，建立完善标准实施后评估调整机制。
　　2．提升服务质量。
　　引导企业学习借鉴先进技术和管理模式，从消费者需求出发，推动服务从“有没有”向“好不好”方向转变，提高服务精细化、专业化水平。依托互联网载体，运用现代服务质量测量评价技术，健全企业服务档案，逐步形成覆盖全国、跨行业、标准统一的征信系统。发布服务质量信息，强化社会监督机制，完善消费者维权和纠纷解决机制。

专栏6 质量提升

实施提升居民生活服务业质量行动计划。鼓励居民生活服务企业建立教学培训基地，推动教育系统将餐饮等服务课程纳入本科院校教育体系，引导企业把提质增效融入经营管理、服务提升的全过程，培育一批具有行业标杆和引领效应的品牌和企业，充分发挥知名企业的引领作用，提升服务质量和水平。建立企业服务质量主体责任制度、服务质量跟踪回访制度和服务质量档案，完善企业服务信息采集制度、服务质量认证体系。定期发布企业服务质量和行业满意度情况，曝光服务质量“黑名单”企业，切实保障消费者的权益。

　　3.传承优秀服务技艺。
　　挖掘优秀服务技艺，支持举办居民生活服务技能大赛，建设独特技能展示平台，建立技艺产权保护目录，培育一批具有优秀服务技能的大师、能工巧匠、服务能手和技术标兵。传承优秀技艺，完善政府、协会、企业多方参与的多层次培训体系，健全从业人员岗位技能培训机制，将优秀传统技艺纳入科研院所的教学活动，有条件的省市可通过建立传承人工作室、教学基地等方式进行“传帮带”，培育精益求精的工匠精神，培养特殊技艺的传承人。创新服务技艺，支持企业运用先进适用技术创新传统技艺，举办体现传统文化、符合现代生活理念的文化节等活动，鼓励创新经营管理模式，充分发挥优秀技艺的品牌价值。
　　（五）以国际化为引领拓展服务空间。
　　1.加强对外开放合作。
　　支持企业“走出去”，加强信息服务，健全风险防范机制，引导居民生活服务企业向境外延伸服务网络，以“一带一路”沿线国家为重点拓展国际市场，培育一批具有国际竞争力的品牌。积极引进国外先进企业，推动有关部门有序放宽家政等居民生活服务准入限制，重点引进管理经验、商业模式和知名品牌，提升利用外资的质量和水平，鼓励居民生活服务领域的跨国公司来华设立总部和功能性机构。推动标准国际化，加大国际标准采标力度，推进我国居民生活服务标准与国际接轨、与国外标准互认，积极参与国际标准制定。
　　2.弘扬中华传统服务。
　　中华传统服务主要涵盖餐饮、民间艺术和民俗、中医、中药、保健、传统手工技艺等领域，蕴含着丰富的民族文化和历史积淀，得到社会广泛认同，具有一定创新发展空间。健全中华传统服务管理体制，完善促进政策，优化企业发展环境，推动对外交流合作，不断提升标准化、信息化、产业化、现代化水平，促进中餐、中医药、中华武术、民间艺术、传统曲艺、传统教育等行业全面协调发展。加强老字号商标保护，在传承传统技艺的基础上，运用现代理念和技术创新服务方式，开发特色服务。力争到2020年，形成区域布局合理、各业态协调发展的格局，培育一批竞争力较强的中华传统服务企业，打造一批享誉海外的中华传统服务品牌。

专栏7 推动国际化发展

实施中华传统服务振兴工程。结合服务贸易创新发展试点工作，会同有关部门制定出台支持政策，建立监管平台，建设“中华传统服务出口基地”，推动中华传统服务企业“走出去”。

实施海外中餐行动计划。鼓励有实力的餐饮企业到国外开设经营网点，推动国内中餐企业与当地餐饮企业开展互利共赢的务实合作，引导海外中餐企业采用国内中餐高水平服务标准，推动中餐纳入世界非物质文化遗产名录，支持餐饮行业中介组织加强国际交流与合作，提升中国文化软实力。

　　（六）以优化环境为保障提升发展动力。
　　1.健全诚信体系。
　　规范居民生活服务企业经营行为，建立开放、公平的市场竞争环境。完善诚信机制，建立以交易信息为基础的市场化综合信用评价机制，鼓励有资质的第三方信用服务机构开展行业信用评价工作，健全信用评价指标，对企业信用、从业人员行为、服务流程、纠纷处理等进行规范；支持行业中介组织建立会员企业信用档案，推动政府向社会机构购买大数据资源和技术服务，加快建立信息共享机制，在行政管理中依法建立以企业信用记录为基础的奖惩制度。开展诚信评价，以统一社会信用代码为基础将居民生活服务企业信用信息纳入全国统一的共享交换平台，形成跨部门、跨地区信用信息网，提供信用信息服务；探索发布信用“红黑榜”，创建诚信示范企业，在全社会形成“守信光荣、失信可耻”的良好氛围。加强诚信监督，加大整治力度，落实企业进货查验、索证索票和质量承诺制度，督促企业履行主体责任；会同工商等部门开展综合执法，提升执法效能，形成纵向到底、横向到边的监管体系；依法统一公开执法信息，为广大群众提供畅通便捷的举报、投诉、申诉和咨询渠道，落实举报投诉奖励措施，引导消费者积极举报投诉，充分发挥举报投诉的监督作用。
　　2.保障供给安全。
　　配合有关部门完善居民生活服务业安全生产法规标准体系，建立安全生产不良记录“黑名单”，做好安全专项治理工作，督促企业强化主体责任，贯彻落实各项安全管理法规、标准、制度和措施，建立健全突发事件应急预案、应对机制，加强安全生产培训，引导企业依法生产经营。

专栏8 优化发展环境

开展诚信示范建设。鼓励企业建立包括用户信息认证、信用等级评价、业务流程保障等内容的信用体系，定期对供应商和员工进行信用评价，建立健全信用信息公开制度。充分利用广播、电视、报刊杂志、互联网等媒介发挥示范企业的引领作用，宣传守信典型案例，公开失信惩戒案例，带动行业信用水平的提升。

开展安全生产宣传。配合安全生产监管部门督促企业完善预防性工作机制、加强源头治理、提高应急处置能力，在广播、电视、报刊和网站加强宣传，利用政务微信、微博、新闻客户端和手机报等方式对企业安全生产敲响警钟，提高企业对安全生产规律的认识，重点宣传好的经验做法，曝光安全隐患，通报企业“黑名单”。

　　**四、**保障措施
　　（一）健全法律法规。
　　加快制修订居民生活服务业法规，明确政府管理职责、服务双方权利与义务、企业经营需满足的安全环保等要求、服务纠纷解决以及违法处罚等相关内容。完善政府部门监管执法体制，将对居民生活服务企业的执法纳入商务综合执法的内容，建立行政管理与执法职能间的衔接机制。
　　（二）强化规划引领。
　　加快推动地方居民生活服务专项规划制定工作，加强专项规划与国家战略和相关规划衔接，并根据国内国际发展形势依法适时调整规划预期目标。支持政策创新，推动地级以上城市在公共服务、用地保障、企业融资、人才培养等方面开展政策试点。加强规划政策宣贯，提高社会认知度和应用性，推动居民生活服务业加快发展。
　　（三）完善政策措施。
　　加快推动居民生活服务业行政审批改革，推动各级政府在土地规划和城乡规划中落实好“新建社区商业和综合服务设施面积占比不低于10%”的政策。会同有关部门贯彻落实好小微企业税收优惠政策，鼓励金融机构加大对小微企业的融资支持。会同有关部门密切跟踪居民生活服务业“营改增”改革后税负变动情况，确保所有行业税负水平只减不增；清理各类基金和收费项目，取消不合理收费，落实居民生活服务企业用水、用电、用气价格方面的政策。研究加大财政支持力度，扩大政府购买服务范围，通过购买服务、股权合作等方式支持各类市场主体增加服务和产品供给。
　　（四）加强人才培训。
　　按照需求导向、覆盖广泛、形式多样的原则，引导行业组织和企业建立全职业生涯、全过程衔接的培训制度。充分发挥各类培训机构、技工院校、行业协会及社会团体的作用，加快建立人才培训示范基地，重点围绕法规标准、职业道德、职业技能、服务流程、经营管理等进行培训，切实提高培训的专业性、有效性和多元性，完善培训、考核、上岗的服务模式，提升服务质量和水平。鼓励各类培训机构和企业开展服务人员培训，对吸纳残疾人等就业困难人员的生活服务市场主体按规定给予相关补贴。
　　（五）做好统计分析。
　　健全居民生活服务典型企业统计监测体系，优化样本结构，完善统计指标，强化数据质量控制，加强运行分析，探索服务消费统计的途径和方法，提高统计数据的准确性和时效性，更好地为宏观决策、行业管理、消费引导提供信息服务和数据支撑。协商统计等部门推动居民生活服务业统计信息共享，构建统计信息共享数据库，推进居民生活服务典型企业统计标准与国际统计标准的衔接。支持社会化信息服务平台建设，建立政府与社会紧密互动的大数据采集机制，鼓励各类平台积极运用大数据技术，加强监测分析，推动基础数据的共享。加强政府公共信息服务，利用商务部门网站及相关新闻媒体，及时发布居民生活服务业运行情况及预测预警信息，更好地指导企业经营、引导居民消费。
　　（六）发挥中介组织作用。
　　支持事业单位、协会在加强统计分析和业务交流、参与项目评估以及人员培训等方面开展工作，帮助企业解决实际困难。支持研究机构、高等院校参与重大战略研究、重大项目的决策咨询和居民生活服务统计监测体系建设，进一步推动国际交流。完善行业自律机制，提高行业规范化发展水平，维护开放、公平、公正的市场竞争环境，保护消费者和企业合法权益，促进居民生活服务业健康有序发展。

© 北大法宝：（[www.pkulaw.com](https://www.pkulaw.com/)) 专业提供法律信息、法学知识和法律软件领域各类解决方案。北大法宝为您提供丰富的参考资料，正式引用法规条文时请与标准文本核对。
欢迎查看所有[产品和服务](https://www.pkulaw.com/helps/2.html)。法宝快讯：[如何快速找到您需要的检索结果？](https://www.pkulaw.com/helps/70.html)[法宝](https://www.pkulaw.com/helps/69.html)V6有何新特色？

扫描二维码阅读原文

**原文链接：**<https://www.pkulaw.com/chl/bd699b986c844f07bdfb.html>
